# Are coveralls required as personal protective equipment during the management of COVID-19 patients?

**DOI:** 10.1186/s13756-021-01017-3

**Published:** 2021-11-27

**Authors:** Jongtak Jung, Kyoung-Ho Song, Hyeonju Jeong, Sin Young Ham, Eu Suk Kim, Hong Bin Kim

**Affiliations:** 1 Department of Internal Medicine, Seoul National University Bundang Hospital, Seoul National University College of Medicine, 82, Gumi-ro173 Beon-gil, Bundang-gu, Seongnam-si, Gyeonggi-do 13620 Republic of Korea; 2 Present Address: Ansung Hospital, Gyeonggi Provincial Medical Center, Ansung, Gyeonggi-do Republic of Korea; 3 Present Address: Department of Internal Medicine, Veterans Health Service Medical Center, Seoul, Republic of Korea

**Keywords:** Severe acute respiratory syndrome coronavirus 2, Coronavirus disease, Personal protective equipment

## Abstract

**Objectives:**

Few studies have investigated the contamination of personal protective equipment (PPE) during the management of patients with severe-to-critical coronavirus disease (COVID-19). This study aimed to determine the necessity of coveralls and foot covers for body protection during the management of COVID-19 patients.

**Methods:**

PPE samples were collected from the coveralls of physicians exiting a room after the management of a patient with severe-to-critical COVID-19 within 14 days after the patient’s symptom onset. The surface of coveralls was categorized into coverall-only parts (frontal surface of the head, anterior neck, dorsal surface of the foot cover, and back and hip) and gown-covered parts (the anterior side of the forearm and the abdomen). Sampling of the high-contact surfaces in the patient’s environment was performed. We attempted to identify significant differences in contamination with severe acute respiratory syndrome coronavirus 2 (SARS-CoV-2) between the coverall-only and gown-covered parts.

**Results:**

A total of 105 swabs from PPEs and 28 swabs from patient rooms were collected. Of the PPE swabs, only three (2.8%) swabs from the gown-covered parts were contaminated with SARS-CoV-2. However, 23 of the 28 sites (82.1%) from patient rooms were contaminated. There was a significant difference in the contamination of PPE between the coverall-only and gown-covered parts (0.0 vs 10.0%, *p* = 0.022).

**Conclusions:**

Coverall contamination rarely occurred while managing severe-to-critical COVID-19 patients housed in negative pressure rooms in the early stages of the illness. Long-sleeved gowns may be used in the management of COVID-19 patients.

**Supplementary Information:**

The online version contains supplementary material available at 10.1186/s13756-021-01017-3.

## Introduction

The World Health Organization (WHO) recommends the rational use of personal protective equipment (PPE) based on the setting, personnel, and type of activity, owing to the recent shortage of PPE globally [1]. The WHO recommends the use of medical masks, goggles or facial shields, gowns, and gloves when providing direct care to patients with coronavirus disease (COVID-19) [1]. However, a discrepancy has been observed between international and local guidelines with regard to the social circumstances [2].

Initially, the Korea Centers for Disease Control and Prevention recommended the use of coveralls and foot covers for protection; however, after several updates in the guidelines, the Center now recommends the use of either a coverall or a water-resistant long-sleeves gown during the management of patients with COVID-19 [2]. Although recent evidence suggests that the transmission of severe acute respiratory syndrome coronavirus 2 (SARS-CoV-2) through contact with fomite is rare [3, 4], there are still concerns regarding viral contamination; thus, many hospitals in Korea still use coveralls with foot covers rather than gowns for protection while managing COVID-19 patients [5].

Research indicates that the PPE of healthcare workers is not contaminated extensively during the management of patients with COVID-19 [6–12]; however, few studies have investigated the contamination of PPE during the management of patients with severe-to-critical COVID-19. Therefore, we conducted this pilot study to determine the necessity of coveralls for body protection during the management of patients with severe-to-critical COVID-19 in the early stages of the illness.

## Methods

### Hospital setting and patients

This study was conducted in nationally designated isolation units consisting of nine single-patient rooms with anteroom of a tertiary-care, university-affiliated medical center in the Republic of Korea [13]. All patients involved in this study were managed in high-level isolated negative pressure rooms [14]. The ventilation rate in the negative pressure room was 20 air changes per hour. Routine room cleaning and disinfection of high contact areas around the patient’s environment were performed daily using sodium hypochlorite. Since the start of the COVID-19 pandemic, the infection control center of our hospital has recommended the use of N95 respirator or powered air purifying respirator (PAPR), goggles or facial shields, gloves, and coveralls with foot covers for healthcare workers when entering isolation units.

Between February 17 and April 19, 2021, patients with severe-to-critical COVID-19 who were within 14 days after symptom onset were included. If supplementary oxygen was required in patients with radiologic pneumonia, they were classified as having severe disease, and patients with severe oxygenation impairment (PaO_2_/FiO_2_ of ≤ 300) were classified as having critical disease according to the WHO classification [15].

### Sample collection, data collection, and analysis

**Fig. 1:**
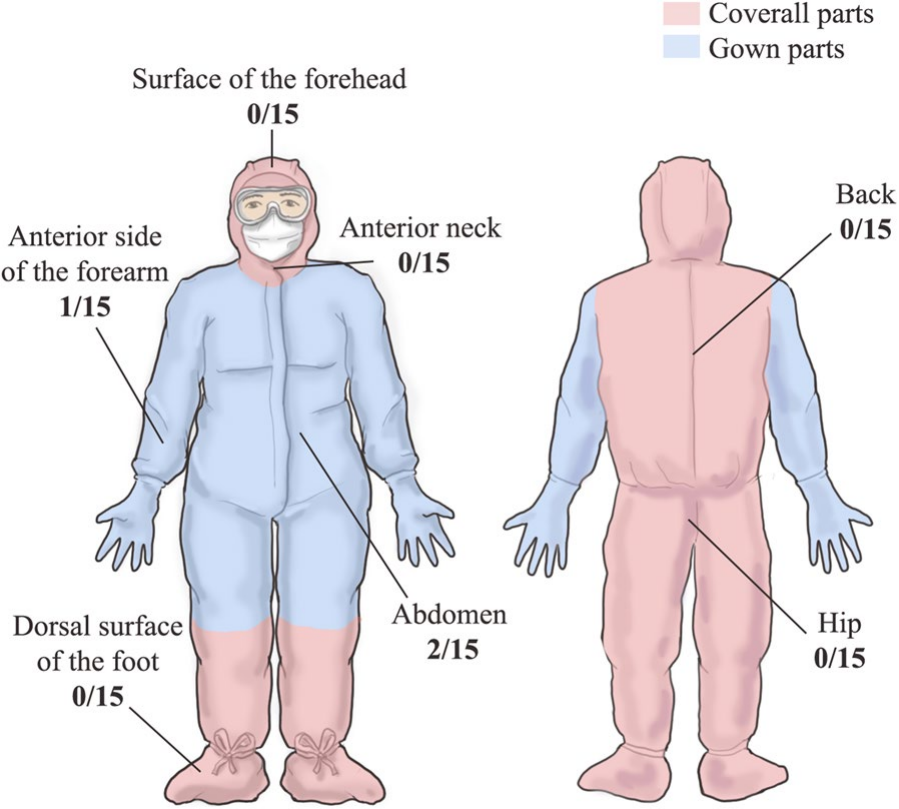
Sampled sites of coveralls and contamination by severe acute respiratory syndrome coronavirus 2. The proportion of contaminated samples to the total collected samples are indicated alongside each sampled site

**Fig. 2:**
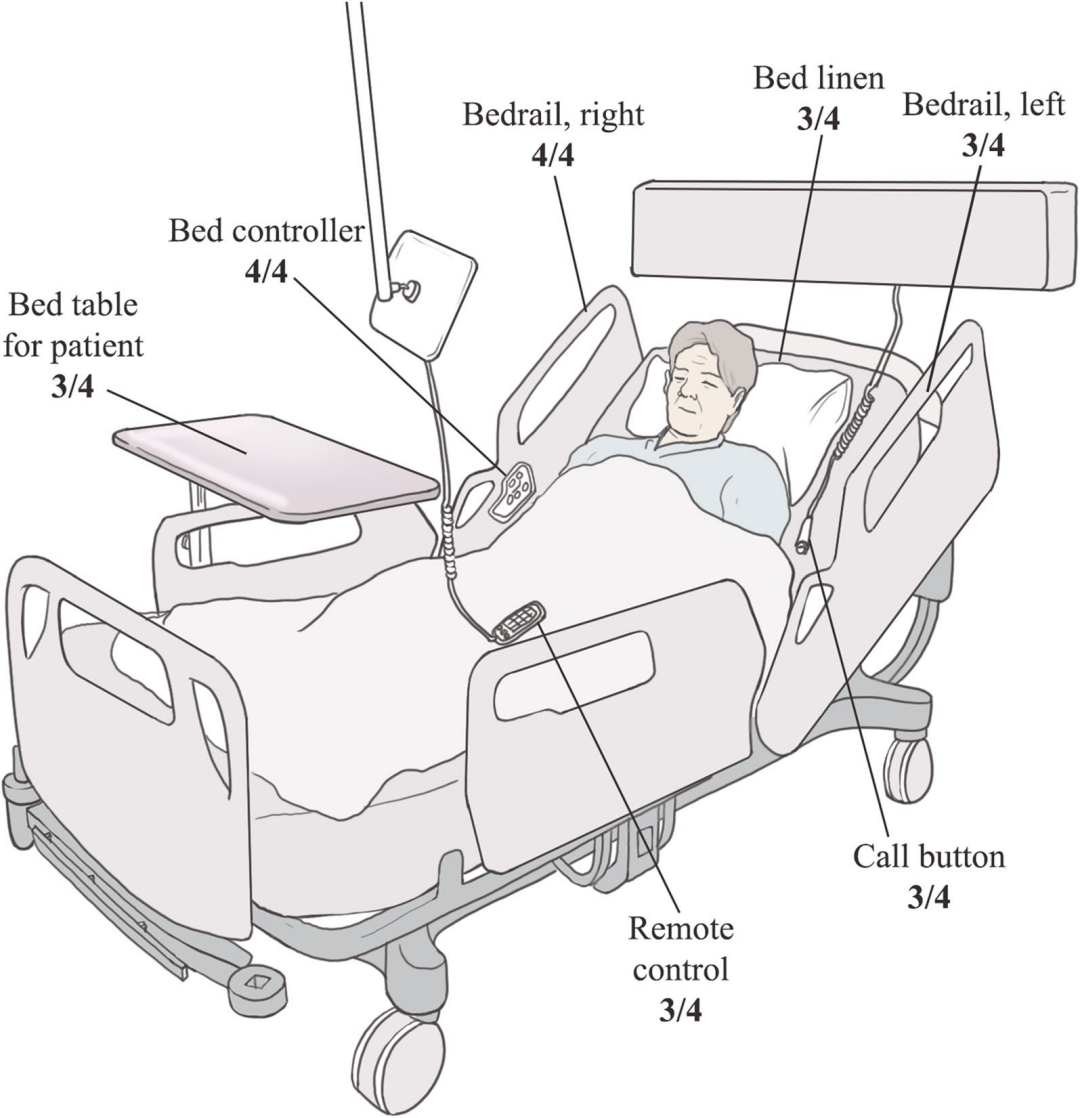
Sampled sites of the environment around the patient and contamination by severe acute respiratory syndrome coronavirus 2. The proportion of contaminated samples to the total collected samples are indicated alongside each sampled site

Samples were acquired from seven sites on the PPE of 15 physicians exiting nine patient rooms. To identify the necessity of coveralls, we collected samples only from the surface of the coveralls, and not from masks, gloves, or goggles. The sampled sites comprised the frontal surface of the head, anterior neck, anterior of the forearm, abdomen, dorsal surface of the foot cover, and back and hip (Fig. 1) [6–12]. To assess environmental contamination, we acquired samples from seven high-contact surfaces of four patient rooms [16]. The environmental sampling sites were the bed linen around the patient’s head, bed controller, both side rails, remote control for the television, call-button, and bed-side table (Fig. 2). Pre-moistened sterile swabs with viral transport medium were used to collect samples in 20 × 20 cm areas, according to the sampling protocol of environmental surfaces by the WHO [16]. Real-time reverse transcriptase-polymerase chain reaction (RT-PCR) targeting E, S, and RdRP/S genes was used to detect SARS-CoV-2 (Allplex™ SARS-COV-2 Assay, Seegene Inc.) [17]. Clinical data, including day of illness, symptoms, disease severity, and RT-PCR results of respiratory specimens, were collected. Activities and the time spent by the physicians in patient rooms were recorded.

Coveralls cover the entire body, including the head, lower legs, and the back side of the body; however, long-sleeved gowns do not cover the head and lower legs, and protection of the back side is compromised owing to its open back design. To examine the necessity of coveralls for body protection, the surface of the coveralls was classified into coverall-only parts (the frontal surface of the head, anterior neck, dorsal surface of the foot cover, and back and hip) and gown-covered parts (the anterior side of the forearm and abdomen) (Fig. 1). Fisher’s exact test was used to identify differences in contamination depending on the parts of the PPE, contact time, symptom onset, and physical activity. A *p*-value of < 0.05 was considered statistically significant.

## Results

A total of 105 swabs from PPEs and 28 swabs from the patient’s environment were collected. The median sampling days from symptom onset was 9 days (range, 2–12 days), the median sampling days from admission was 3 days (range, 1–7 days), and the median contact time with patients was 20 min (range, 10–30 min). Activities commonly performed by physicians in the patient’s room were general care, physical examination, and acquisitions of respiratory samples. AGPs, such as intubation, suctioning of airway, or nebulizer therapy, were performed in five cases. Of the PPE swabs, only three (two from the abdomen and one from the forearm) were contaminated with SARS-CoV-2. All swabs from the coverall-only parts tested negative. There was a significant difference in the contamination of PPE between the coverall-only and gown-covered parts (0.0 vs 10.0%, *p* = 0.022). There were no significant differences in the contamination of PPEs according to symptom onset, contact time, or aerosol producing procedures (Additional file 1: Table 1). The detailed clinical information regarding the included patients and contamination of PPE are shown in Table 1. Of the patient’s environment swabs, 20 of the total 28 sites (82.1%) were contaminated. The detailed contamination sites and cycle threshold of the environment are presented in Table 2.

**Table 1:** Clinical characteristics of the included patients and contamination of personal protective equipment according to the type of activity and contact time

PPE	Patient	Type of oxygen delivery	PF ratio	Sampling days from admission	Sampling days from symptom onset	Type of activity	Aerosol generating procedures	Contact time (min)	Ct value of respiratory samples	Sites of PPE contamination (Genes, Ct value)
1	A	HFNC	73	5	10	Examination, general care	No	20	22.52	ND
2	B	HFNC	125	3	11	Examination, general care	No	10	20.13	ND
3	A	HFNC	73	6	11	Examination, general care, acquisition of nasopharyngeal and lower respiratory sample	No	10	26.54	ND
4	A	MV	72	7	12	Examination, intubation, acquisition of nasopharyngeal and lower respiratory sample, suctioning of airway	Yes (intubation, suctioning of airway)	20	26.54	ND
5	C	HFNC	94	1	9	Examination, general care, acquisition of nasopharyngeal and lower respiratory sample	No	30	19.65	ND
6	C	HFNC	94	1	9	Examination, general care	No	30	19.65	ND
7	C	HFNC	69	2	10	Examination, general care	No	15	15.56	Abdomen (RdRP/S 38.62, E 38.17, N ND)
8	D	NP	243	2	2	Examination, general care, suctioning of airway	Yes (suctioning of airway)	15	13.88	ND
9	C	MV	92	3	11	Examination, general care, acquisition of nasopharyngeal and lower respiratory sample, suctioning of airway	Yes (suctioning of airway)	20	22.19	Forearm (RdRP/S ND, E 37.91, N ND)
10	D	HFNC	73	7	7	Examination, general care, acquisition of nasopharyngeal and lower respiratory sample, suctioning of airway	Yes (suctioning of airway)	20	24.23	ND
11	F	HFNC	75	3	6	Examination, general care, acquisition of nasopharyngeal and lower respiratory sample	No	30	22.63	ND
12	G	HFNC	150	2	5	Examination, general care, acquisition of nasopharyngeal and lower respiratory sample	No	20	16.87	ND
13	G	HFNC	113	5	8	Examination, general care, acquisition of nasopharyngeal and lower respiratory sample, nebulizer	Yes (nebulizer)	20	22.09	ND
14	H	HFNC	108	5	10	Examination, general care, acquisition of nasopharyngeal and lower respiratory sample	No	20	19.10	ND
15	I	HFNC	110	2	8	Examination, general care, acquisition of nasopharyngeal and lower respiratory sample	No	30	21.43	Abdomen (RdRP/S 37.91, E ND, N ND)

*PPE* personal protective equipment; *PF ratio* the ratio of arterial oxygen partial pressure to fractional inspired oxygen; *Ct* cycle threshold; *HFNC* high flow nasal canula; *MV* mechanical ventilation; *ND* not detected

**Table 2:** Severe acute respiratory syndrome coronavirus 2 contamination in the patient’s environment

Patient	Sampling days from admission	Sampling days from symptom onset	Environmental contamination (Ct value)						
			Bed linen	Bed controller	Bedrail, right	Bedrail, left	Remote control	Call button	Bedside table
F	3	6	ND	E (33.87) RdRP/S (34.19) N (32.09)	E (38.15) RdRP/S (38.05) N (38.11)	ND	ND	E (38.06) RdRP/S (38.64) N (37.90)	E (38.30) RdRP/S (39.03) N (ND)
G	2	5	E (35.50) RdRP/S (36.60) N (35.02)	E (37.59) RdRP/S (37.75) N (35.01)	E (35.15) RdRP/S (35.17) N (33.64)	E (34.94) RdRP/S (37.61) N (35.60)	E (33.38) RdRP/S (35.19) N (33.22)	E (33.82) RdRP/S (34.37) N (34.08)	E (37.94) RdRP/S (ND) N (37.17)
H	5	10	E (31.25) RdRP/S (31.40) N (32.23)	E (35.31) RdRP/S (35.14) N (34.32)	E (36.80) RdRP/S (37.99) N (36.70)	E (36.99) RdRP/S (35.71) N (37.03)	E (36.26) RdRP/S (35.64) N (37.50)	ND	E (35.70) RdRP/S (35.66) N (36.29)
I	2	8	E (38.22) RdRP/S (36.64) N (ND)	E (35.99) RdRP/S (37.55) N (35.36)	E (35.84) RdRP/S (36.17) N (37.11)	E (35.73) RdRP/S (36.93) N (36.34)	E (34.36) RdRP/S (35.84) N (33.85)	E (34.59) RdRP/S (34.01) N (35.79)	ND

*Ct* cycle threshold; *ND* not detected

## Discussion

This study aimed to determine the necessity of coveralls for protecting the body while managing patients with severe-to-critical COVID-19 during early stages of the illness. Our results revealed that coverall contamination rarely occurred during the management of patients with severe-to-critical COVID-19 in the early stages of the illness, although contamination of the patient’s environment was common. In particular, the coverall-only parts were not contaminated.

These findings are consistent with the results of previous studies. In previous studies, PPE contamination was not observed during the management of asymptomatic or mild COVID-19 patients [8–10]. In some studies [6, 7, 11], some parts of the PPE, even on the top of the head or foot covers, were contaminated during the routine care of patients over an extended period (4 h) [7], but most parts of the PPE were not contaminated, and the viability of virus was not confirmed.

In previous studies, environmental contamination in isolation rooms varied from 1.4% to 100% [3], but extensive contamination of the environment around the patients was identified in our study despite routine cleaning and disinfection. The viral load of the patients and the severity of COVID-19 have been shown to be positively correlated with environmental contamination [18–22]. Contamination could have been extensive because we collected samples from the environment of the patients with severe-to-critical COVID-19 who were in the early stage of illness. This suggests that more aggressive disinfection of the patient’s environment should be considered for areas that are in high contact in the early stages of illness.

Although the environment of the patient rooms was widely contaminated, it was found that the contamination of PPE rarely occurred if the contact time with patients was relatively short (≤ 30 min). Contamination of the coveralls was identified only in the gown-covered parts and it was statistically significant. This suggests that a long-sleeved gown would be adequate to protect the body from contamination with SARS-CoV-2. Because contamination rarely occurred, significant differences of contamination according to symptom onset, contact time, or type of activities were not identified.

Donning coveralls requires more time than donning long-sleeved gowns, which makes it difficult to respond to emergency situations. Furthermore, because coveralls cover the entire body, they can induce heat stress more easily than gowns, causing dehydration and exhaustion, which may influence performance [23]. In addition, healthcare workers are not familiar with coveralls, and contamination occurs frequently during the doffing process, indicating the requirement of regular training [24, 25]. Inadequate and overuse of PPEs shown in mass media can cause excess fear in the general public, leading to social issues, such as mental health problems and stigma [26–28]. Considering the disadvantages of coveralls and a global shortage of PPEs, protective clothing should be reasonably used according to the risk of contamination.

Because contamination of PPE, particularly the coverall-only parts, rarely occurred during the short-term management of COVID-19 patients regardless of disease severity, it can be expected that long-sleeved gowns could provide sufficient protection from SARS-CoV-2 contamination. Recent evidence suggests that the dominant route of viral transmission is via the respiratory tract by droplet or aerosol, and that transmission through contact with fomite is rare [3, 4, 29]; therefore, the use of long-sleeved gowns (with gloves, mask, goggles, or facial shields) in conjunction with hand hygiene and routine cleaning and disinfection of the hospital environment may protect healthcare workers from infection with SARS-CoV-2 during the management of patients with COVID-19.

This study has some limitations. First, although several studies that have identified the contamination of PPEs have been published, and one study attempted to validate the sampling method [6], to date, the sampling method has not been standardized and its sensitivity and specificity have not been identified, indicating a possibility of minor contamination of PPEs. However, in our study, PPE contamination was rarely identified, in contrast to the patient’s environment, suggesting that the contamination on the PPE surface may be much less than that of the environment. It would be unlikely that such a small amount of the virus could cause infection among healthcare workers. Second, although we acquired samples from PPE in various situations, including AGPs, the sample size was relatively small, and these results cannot be extrapolated to the case of managing patients at high risk of airborne transmission. Third, as this study was conducted only in isolated negative pressure rooms with high ventilation rates, further studies are needed to identify contamination of PPE when managing patients admitted to general wards without negative pressure. The deposition of viral particles onto environmental surfaces is affected by airflow and ventilation [30]; thus, it is expected that environmental contamination would be lower in negative pressure rooms. In one study, environmental contamination in negative pressure rooms was less than that in neutral pressure rooms [21]; thus, there is a possibility that the contamination of PPE would be more common when managing patients with COVID-19 in general wards without negative pressure. Fourth, there may be a higher chance of PPE contamination for nurses who stay in isolation rooms for longer durations and are in closer contact with patients or for other healthcare workers who perform different activities with physicians; therefore, further studies are required. However, in our study, contamination of coveralls rarely occurred despite various types of activities performed by physicians; thus, contamination of coveralls is not likely to occur widely even for other healthcare workers if they stayed in patient rooms for a relatively short duration (within 30 min).

## Conclusion

In the present study, we found that coverall contamination rarely occurred while managing severe-to-critical COVID-19 patients admitted to negative pressure rooms during early stages of the illness. Long-sleeved gowns could suffice when managing COVID-19 patients.

## Supplementary Information

**Additional file 1.** Contamination of protective personal equipment according to symptom onset, contact time, and aerosol producing procedures.

## Data Availability

The datasets used and/or analyzed during the study are available from the corresponding author on reasonable request.

## Abbreviations

WHO: World Health Organization
PPE: Personal protective equipment
COVID-19: Coronavirus disease
AGPs: Aerosol generating procedures
SARS-CoV-2: Severe acute respiratory syndrome coronavirus 2
RT-PCR: Reverse transcriptase-polymerase chain reaction
