# Supplementary material for: Are coveralls required as personal protective equipment during the management of COVID-19 patients?
Source: Antimicrob Resist Infect Control. 2021 Nov 27;10:164. doi: 10.1186/s13756-021-01017-3 (PMC8626720; doi:10.1186/s13756-021-01017-3)
Supplement: Supplementary file 1 — Additional file 1. Contamination of protective personal equipment according to symptom onset, contact time, and aerosol producing procedures. [file 13756_2021_1017_MOESM1_ESM.docx]

**Supplementary Table 1. Contamination of protective personal equipment according to symptom onset, contact time, `and aerosol producing procedures**

|  | **Symptom onset ≤ 7 days** | **Symptom onset > 7 days** | **p-value** |
| --- | --- | --- | --- |
| **Number of contaminated parts of PPE** | 0 (0.0%) | 3 (3.6%) | 1.000 |
|  | **Contact time ≤ 10 min** | **Contact time > 10 min** | **p-value** |
| **Number of contaminated parts of PPE** | 0 (0.0%) | 3 (3.3%) | 1.000 |
|  | **AGP** | **No AGP** | **p-value** |
| **Number of contaminated parts of PPE** | 2 (2.9%) | 1 (2.9%) | 1.000 |

PPE, personal protective equipment; AGP, Aerosol generating procedure
